# Para-Anastomotic Pseudoaneurysms as a Long-Term Complication After Surgical Treatment of Peripheral Artery Disease: Clinical Characteristics and Surgical Treatment

**DOI:** 10.3390/biomedicines12122727

**Published:** 2024-11-28

**Authors:** Julia Łacna, Michał Serafin, Dorota Łyko-Morawska, Julia Szostek, Dariusz Stańczyk, Iga Kania, Magdalena Mąka, Waclaw Kuczmik

**Affiliations:** Department of General Surgery, Vascular Surgery, Angiology and Phlebology, Faculty of Medical Sciences in Katowice, Medical University of Silesia, 45-47 Ziołowa Street, 40-635 Katowice, Poland; lacnaj@onet.pl (J.Ł.); s81394@365.sum.edu.pl (J.S.); s80989@365.sum.edu.pl (I.K.); s81151@365.sum.edu.pl (M.M.);

**Keywords:** peripheral arterial disease, para-anastomotic aneurysms, surgical treatment

## Abstract

**Background:** Peripheral arterial disease (PAD) is becoming an increasingly prevalent clinical issue, leading to a growing number of patients requiring surgical interventions. Consequently, there is an increasing occurrence of para-anastomotic aneurysms as late complications following primary treatment for PAD. These aneurysms typically arise at the sites of graft implantation and necessitate individualized management strategies based on factors such as location, size, and the patient’s overall condition. **Materials and Methods:** This five-year retrospective study, conducted at a single center, aimed to evaluate the anatomical location, clinical presentation, diagnostic methods, and management strategies for 55 patients treated for femoral and popliteal artery para-anastomotic pseudoaneurysms of the lower limb between January 2018 and June 2024. Treatment approaches were determined based on aneurysm size, the extent of atherosclerosis, and the patient’s surgical risk. This study analyzed patient demographics, surgical techniques, postoperative complications, and aneurysm characteristics. **Results:** Most pseudoaneurysms occurred between 6 and 10 years after the primary procedure. The most common surgical intervention was aneurysmectomy with graft interposition, performed in 46 patients (83.64%), followed by aneurysmectomy with extra-anatomical bypass in 6 patients (10.91%), and endovascular repair (EVAR) in 3 patients (5.45%). Early postoperative complications occurred in 16.36% of patients. The 12-month freedom from graft stenosis was 87.23%, and freedom from anastomotic aneurysm recurrence at 12 months was 100%. **Conclusions:** This study highlights the critical need for individualized treatment strategies and ongoing surveillance in managing lower-limb para-anastomotic pseudoaneurysms, particularly given the prevalence of lower-limb pain and the high occurrence of such in the common femoral artery. The favorable long-term graft patency rates observed suggest that aneurysmectomy with graft interposition is an effective intervention, reinforcing its role as the primary approach within this patient population.

## 1. Introduction

Peripheral arterial disease (PAD) is a prevalent and increasingly common condition worldwide, with an estimated 230 million individuals affected. Globally, PAD ranks third in morbidity among atherosclerotic diseases, following coronary heart disease and stroke [1]. Over the past decade, the global burden of PAD has risen by more than 25%, with the greatest increase observed in low- and middle-income countries [2].

Early diagnosis and timely intervention are essential for improving patient outcomes and preventing serious complications such as limb ischemia and amputation. According to the European Society for Vascular Surgery (ESVS) guidelines, the primary treatment for PAD focuses on lifestyle modifications and pharmacological management. However, in severe cases with symptomatic disease, invasive revascularization is required. This may involve percutaneous angioplasty, with or without stenting, endarterectomy, or open surgical bypass [3]. While open surgical bypass is often successful in treating PAD, late complications can substantially affect patient outcomes. One such complication is the development of a para-anastomotic pseudoaneurysm, which can arise at the site of the vascular anastomosis following the procedure. These pseudoaneurysms represent a significant postoperative challenge and require careful monitoring and management to mitigate their impact on long-term survival and quality of life [3].

Pseudoaneurysms are characterized by incomplete vessel dilation, confined only to the adventitia, which distinguishes them from true aneurysms, where the dilation involves all layers of the vessel wall [4]. Historically, incidences of para-anastomotic pseudoaneurysms were reported to be 10% over a 10-year period in patients with vascular anastomosis [5]. However, more recent studies [6] suggest that their frequency ranges from 2.2% to 3.6% at 5 and 10 years, respectively.

Due to their infrequent detection during physical examination, the advancement of imaging diagnostics is essential for more effective identification of these pathologies [7]. Ultrasonography and duplex Doppler ultrasonography, both characterized by high sensitivity and specificity, are considered the first-line imaging tools in pseudoaneurysm diagnosis [8]. Duplex ultrasound is highly effective for diagnosing peripheral artery aneurysms in the lower limbs, as the superficial location of arteries allows for excellent imaging quality. This noninvasive, accessible, and affordable technique reliably provides detailed information about the structure of inflow and outflow vessels, aneurysm size, adjacent arteries, and the presence of intraluminal thrombus. The Doppler component is especially helpful in identifying areas of stenosis, occlusion, arteriovenous connections, and related venous complications like thrombosis or compression [9]. However, it is important to note that the effectiveness of these techniques largely depends on the experience of the operator. Computed tomography angiography (CTA) is crucial for assessing the extent of vascular changes, making therapeutic decisions, and planning treatment [8,10]. The presence of a qualified radiologist is essential in these cases. CTA is the recommended diagnostic method for evaluating lower-extremity pseudoaneurysms prior to surgical intervention [10,11,12].

According to the guidelines of the ESVS, the treatment of femoral artery aneurysms and pseudoaneurysms should primarily be based on open surgery [8,13]. Symptomatic aneurysms/pseudoaneurysms are treated with open methods regardless of their size, while asymptomatic femoral artery aneurysms/pseudoaneurysms require treatment if they exceed 2.5 cm in diameter, as do popliteal artery aneurysms/pseudoaneurysms if they exceed 2 cm. These procedures are particularly recommended in cases of rapid aneurysm expansion, rupture, infection, distal ischemia, or peripheral neuropathy, as they allow for complete aneurysm removal and provide better control over the treatment process [14,15,16]. Additionally, the presence of scarring or vascular calcifications resulting from prior endovascular interventions, which significantly hinders endovascular access, may also suggest the choice of open surgery. Considering the variability of lower-limb anastomotic pseudoaneurysms and the range of available treatment options, management should be individualized based on the pseudoaneurysm’s etiology, location, and size, along with the patient’s overall clinical condition and expected postoperative quality of life.

This study is focused on delineating the characteristics of para-anastomotic pseudoaneurysms after the primary surgical bypass for PAD in the lower limb and assessing the outcomes of invasive treatment across perioperative, short-term, and long-term periods. The investigation utilizes data sourced from the Department of General Surgery, Vascular Surgery, Angiology, and Phlebology at the Medical University of Silesia, Katowice, Poland.

## 2. Materials and Methods

### 2.1. Study Design and Population

This retrospective analysis included all patients managed for femoral (common, deep, and superficial) or popliteal artery aneurysms within the Department of General Surgery, Vascular Surgery, Angiology, and Phlebology at the Medical University of Silesia in Katowice, Poland, from January 2018 to June 2024. A comprehensive review of the electronic medical records was conducted for 131 lower-limb true aneurysm and pseudoaneurysm cases, focusing solely on those undergoing primary treatment for anastomotic pseudoaneurysm that developed after the primary treatment of PAD with surgical bypass. Patients who received treatment for true aneurysms, pseudoaneurysms of alternative etiologies (post-traumatic pseudoaneurysm, iatrogenic pseudoaneurysm), or recurrent pseudoaneurysms were excluded from the analysis.

The group with anastomotic pseudoaneurysms consisted of 55 (41, 74.55% men; 14, 25.45% female) patients with a mean age of 67.45 (54–83, SD: 6.89).

### 2.2. Anastomotic Pseudoaneurysm Diagnostic Criteria

**Figure 1:**
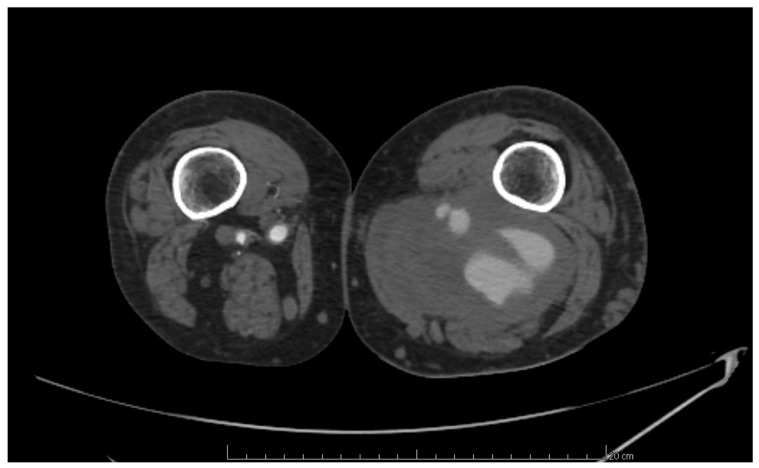
Para-anastomotic pseudoaneurysm of popliteal artery.

The diagnosis of anastomotic aneurysms was performed using computed tomography angiography (CTA), following the European Society for Vascular Surgery Clinical Practice Guidelines [8]. These pseudoaneurysms were identified in patients with arterial dilation involving only one or two layers of the arterial wall, surrounded by periarterial connective tissue. Additionally, the arterial dilation was situated adjacent to the arterial anastomosis site (Figure 1).

Invasive treatment was conducted in the case of symptomatic anastomotic pseudoaneurysms as well as asymptomatic femoral artery or popliteal artery anastomotic pseudoaneurysms with a diameter over 25 mm or 20 mm, respectively.

### 2.3. Treatment Methods

The procedure of aneurysmectomy with graft interposition was performed in situations where minimal atherosclerosis affected the arterial segments adjacent to the aneurysm sac, and blood flow remained adequate. This surgical intervention was deemed suitable when atherosclerotic involvement was limited, allowing for the preservation of vascular integrity and ensuring proper circulation.

Aneurysmectomy combined with surgical bypass was employed in cases where severe atherosclerosis in the arterial segments before and after the aneurysm sac was found. This combined approach was necessary for pseudoaneurysms and for arteries severely affected by atherosclerosis, which compromised blood circulation.

In the case of patients with acute limb ischemia treated with an aneurysmectomy with a graft interposition or extra-anatomical bypass, simultaneous surgical thrombectomy was performed.

Endovascular treatment was utilized for high-risk patients who had contraindications for open surgery. This minimally invasive procedure was selected for individuals unsuited for traditional open surgery due to factors such as advanced age, comorbidities, or other medical conditions that heightened the risk of surgical complications. Endovascular treatment allowed effective management of the pseudoaneurysm while minimizing overall risk to the patient.

During the endovascular treatment, patients with acute limb ischemia underwent simultaneous mechanical thrombectomy.

The SilverGraf (B. Braun Melsungen AG, Berlin, Germany) or the Gelsoft™ Plus Vascular Prosthesis (Terumo Aortic, Inchinnan, UK) was used during open surgical procedures. The choice of vascular prosthesis was determined by the lead vascular surgeon overseeing the procedure. For endovascular treatment, a GORE VIABAHN VBX Balloon Expandable Endoprosthesis (W. L. Gore & Associates, Flagstaff, AZ, USA) was employed.

Postoperative complications were classified as any complications that occurred during a patient’s hospitalization or within 30 days following the surgical procedure. Reoperation was defined as any additional surgical interventions required due to these postoperative complications occurring within the same timeframe.

In contrast, late complications were those that arose more than 30 days after the surgical procedure. Similarly, late reoperation referred to any subsequent surgical procedures necessitated by complications that developed after this 30-day period.

### 2.4. Term Definitions

The total duration of hospitalization was calculated from the date of admission to the date of discharge, while the duration of postoperative hospitalization specifically referred to the time interval between the day of surgery and the day of discharge.

Follow-up was defined as the last recorded visit of the patient to the ward or clinic. Overall survival (OS) was assessed from the date of the surgical procedure until either the date of death or the date of the last contact with the ward or clinic.

Graft stenosis was characterized as a narrowing of the graft exceeding 75%, indicated by a more than 3.5-fold increase in peak systolic velocity, as determined by duplex ultrasound or angiographic assessment.

### 2.5. Data Analyzed in the Study

This study analyzed various parameters, including the general characteristics of patients (such as age, gender, comorbidities, and clinical symptoms), the type and duration of surgery, the incidence of postoperative complications, reoperations, mortality rates, and length of hospitalization, as well as the localization, diameter of the aneurysm, and follow-up data.

General patient characteristics, including age, gender, comorbidities, and clinical symptoms, were retrieved from the medical records in our department. Information regarding the incidence of postoperative complications, such as graft stenosis and the need for reoperations, was also sourced from these records.

Data concerning the location of the aneurysms, along with the duration and nature of the surgical procedures, were derived from the surgical descriptions documented for each patient. The diameter of the aneurysm was measured using computed tomography angiography (CTA) results for each patient.

Follow-up information, including late complications (notably graft stenosis), late reoperations, and mortality rate, was gathered from the patients’ medical histories within the surgical clinic and/or the department.

### 2.6. Statistical Analysis

Statistical analysis was conducted using Statistica^®^ software version 13.3 (StatSoft, Tulsa, OK, USA, 2013). Qualitative variables were presented as absolute values and percentages with contingency tables used to indicate group sizes. For quantitative variables, ranges, means, standard deviations, or medians with interquartile ranges were reported. The Shapiro–Wilk test was employed to assess the statistical distribution among the study participants. Overall survival, graft stenosis and pseudoaneurysm recurrence analyses were carried out using the Kaplan–Meier estimator. A *p*-value of less than 0.05 was deemed statistically significant.

## 3. Results

### 3.1. Demographic and Descriptive Data of the Patients

Within the entire cohort, 49 (89.09%) patients had comorbidities. The most prevalent comorbidity was arterial hypertension (38; 69.09%), followed by general atherosclerosis (26; 47.27%) and coronary artery disease (19; 34.55%). In total, 30 (54.55%) patients had a history of smoking, while 21 (38.18%) were current smokers. Clinical symptoms were present in 34 (61.82%) patients, with the most common being lower-limb pain (19; 34.55%), followed by intermittent claudication (9; 16.36%) (Table 1).

### 3.2. Characteristics of Anastomotic Aneurysms

**Figure 2:**
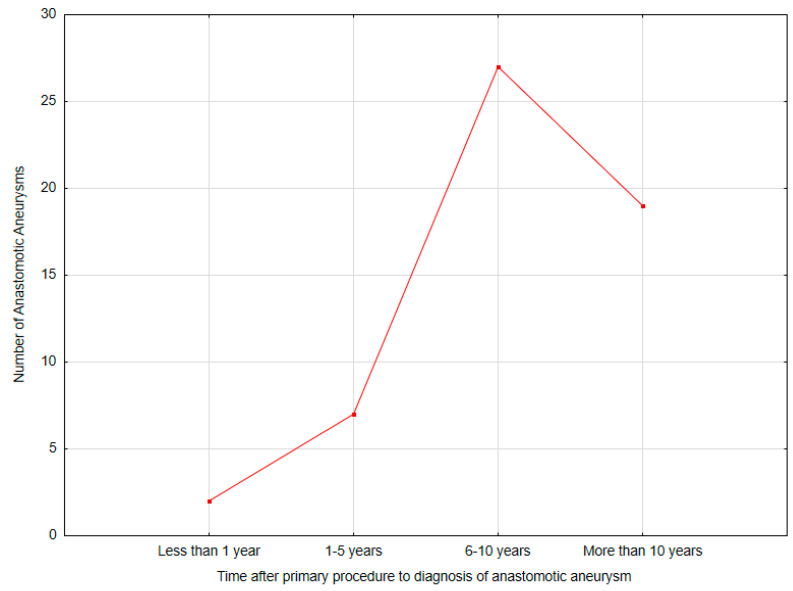
Time intervals between the primary procedure and the diagnosis of anastomotic pseudoaneurysm (Statistica^®^, 13.3, StatSoft).

Most anastomotic pseudoaneurysms occurred after the primary aortobifemoral bypass (36; 65.46%). The median time of the occurrence of anastomotic pseudoaneurysm after the primary procedure was 8.78 (0.21–25.24, IQR: 8.70) years. Most of the anastomotic pseudoaneurysms occurred between 6 and 10 years after the primary procedure (27; 49.09%) The majority of anastomotic pseudoaneurysms occurred in the common femoral artery (50; 90.91%). The median aneurysm size in CTA was 38 (18–260, IQR: 24) mm (Table 2) (Figure 2).

### 3.3. Perioperative Data and Short-Term Outcomes

The median duration of the procedure was 150 min (55–365, IQR: 60). Intraoperative blood loss was less than 400 mL in 52 patients (94.55%). Red blood cell transfusions were required in two patients (3.64%), and fresh frozen plasma transfusion was necessary in one patient (1.82%).

Aneurysmectomy with graft interposition was the most commonly performed procedure, completed in 46 patients (83.64%). This was followed by an aneurysmectomy with extra-anatomical bypass in six patients (10.91%). The median length of hospitalization was 8 days (4–100, IQR: 4), with a post-procedure hospitalization of 4 days (2–90, IQR: 4).

Early postoperative complications were observed in nine patients (16.36%), with the most prevalent being acute limb ischemia (3; 5.45%) and surgical site infection (3; 5.45%), followed by myocardial infarction (2; 3.64%) and hematoma (2; 3.64%). Reoperation was required in five patients (11.11%) due to acute limb ischemia (3; 5.45%) and hematoma (2; 3.64%). There were no in-hospital deaths (Table 3).

### 3.4. Patients’ Long-Term Outcomes After Anastomotic Aneurysm Treatment

Patients’ follow-up time at the clinic was a median of 16.5 (1–67, IQR: 33) months. During this period, 17 (30.91%) late complications occurred. The most common was acute limb ischemia (4; 7.27%), followed by graft infection and anastomotic pseudoaneurysm recurrence (4; 7.27% both). Late reoperations were needed in 10 (18.18%) patients due to anastomotic pseudoaneurysm recurrence (4; 7.27%), acute limb ischemia (4; 7.27%), and graft infection (2; 3.64%). Additionally, in the follow-up period, 6 (10.91%) deaths were observed due to sepsis related to graft infection (4; 7.27%) and myocardial infarction (2; 3.64%) (Table 4).

#### 3.4.1. Graft Patency

**Figure 3:**
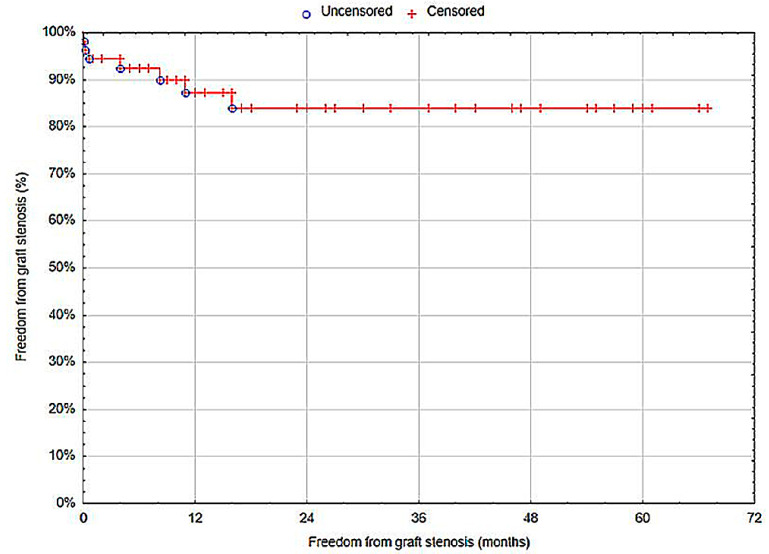
Freedom from graft stenosis (Statistica^®^, 13.3, StatSoft).

Twelve months after the treatment of an anastomotic aneurysm, the graft patency (freedom from graft stenosis) was 87.23% (standard error (SE) = 4.95%) (Figure 3).

#### 3.4.2. Anastomotic Pseudoaneurysm Recurrence

**Figure 4:**
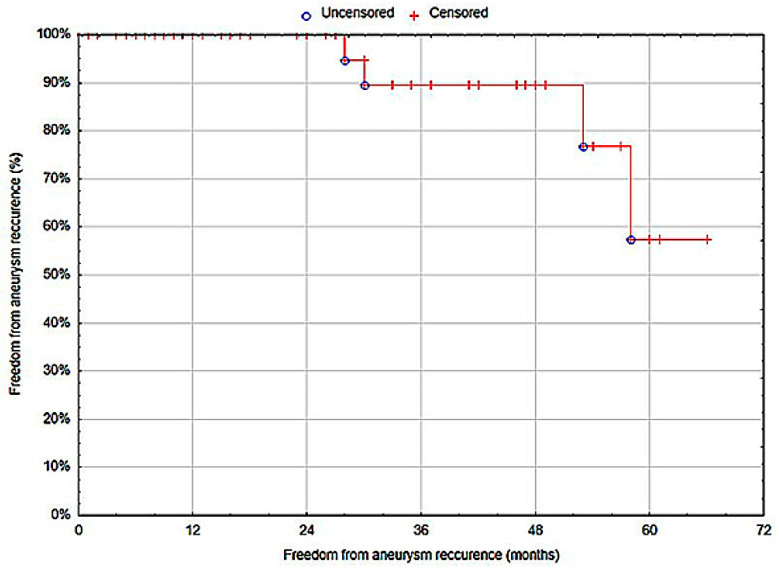
Freedom from anastomotic aneurysm recurrence (Statistica^®^, 13.3, StatSoft).

The freedom from anastomotic aneurysm recurrence at 12 months was 100% (SE: 0%) (Figure 4).

#### 3.4.3. Overall Survival

**Figure 5:**
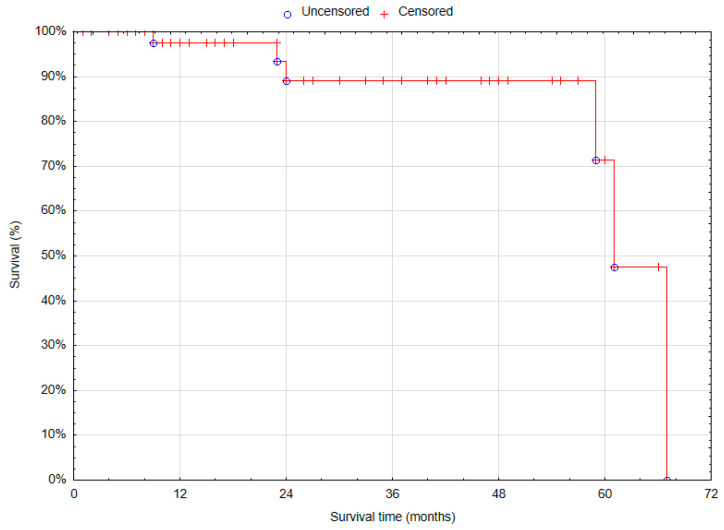
Overall survival in patients treated for anastomotic aneurysms (Statistica^®^, 13.3, StatSoft).

The overall 12-month survival after the treatment of anastomotic aneurysms was 97.5% (SE = 2.46%) (Figure 5).

## 4. Discussion

This study analyzed the demographics, clinical manifestations, procedural outcomes, and long-term follow-up of patients with lower-limb para-anastomotic pseudoaneurysms. The cohort was predominantly male, with 41 men (74.55%) compared to 14 women (25.45%). The primary presenting symptoms included lower-limb pain (34.55%), intermittent claudication (16.36%), and acute limb ischemia (5.45%). A majority of the pseudoaneurysms were located in the common femoral artery (90.91%), followed by the popliteal artery (5.45%), with most pseudoaneurysms manifesting between 6 and 10 years (49.09%) after the primary procedure. Aneurysmectomy was the most common procedure (83.64%), followed by aneurysmectomy with extra-anatomical bypass (10.91%), while endovascular repair was the least common (5.45%). Long-term follow-up revealed acute limb ischemia, graft infection, and anastomotic aneurysm recurrence as the most frequent late complications (7.27% each). It should be noted that all cases of acute limb ischemia or anastomotic pseudoaneurysm recurrence necessitated reoperation (7.27% both), while graft infections were associated with mortality in all cases (7.27%).

The primary symptom reported upon admission was lower-limb pain, occurring in nineteen patients (34.55%). Intermittent claudication was observed in nine patients (16.36%). Furthermore, four patients (7.27%) reported ulceration, four others (7.27%) presented with aneurysm rupture, and three patients (5.45%) developed acute lower-limb ischemia. The frequency of these symptoms in other publications is as follows: lower-limb pain (12.5–41%), intermittent claudication (3.5%–12.8%), aneurysm rupture (2.6–29.9%), and acute lower-limb ischemia (7.7–19.5%) [10,17,18].

It has been noted that the frequency of anastomotic pseudoaneurysm diagnosis increases with time after the primary surgery. In the studied group, anastomotic pseudoaneurysms were diagnosed in two patients (3.64%) within the first year, in seven patients (12.73%) within 1–5 years after surgery, while the majority of cases were diagnosed between 6 and 10 years after the initial procedure (27 cases, 49.09%). A significant number of pseudoaneurysms were also detected more than 10 years postoperatively (19 cases, 34.55%). These numbers differ significantly from the data reported by Marković et al., who described pseudoaneurysm complications occurring within 1 year in 22 patients (26.3%), between 2 and 5 years in 35.5%, between 6 and 10 years in 27.6%, and between 10 and 12 years in 12.6% of cases [17]. In Marković et al.’s study, the incidence of pseudoaneurysms during the first postoperative year was substantial, contrasting with our findings. It is worth noting that in their study, 24.7% of pseudoaneurysm cases were associated with graft infection, which may have contributed to the high detection rate in the first year post-operation. Furthermore, the study was conducted between 1991 and 2002, meaning that both surgical techniques and materials used during that period differed from contemporary standards. The trend of increasing pseudoaneurysm frequency with time after surgery may be related to prolonged exposure to arterial pressure waves or other mechanical factors acting on the anastomosis, leading to its failure. The literature also points to graft infection as a predisposing factor for pseudoaneurysm development [7]. Additionally, tissue growth between the graft and the inguinal ligament prevents graft displacement over the ligament during hip movement, which can lead to anastomotic leakage and, consequently, the formation of a pseudoaneurysm [17]. Mechanical stress at the anastomotic site is a significant factor in the development of pseudoaneurysms. One primary contributor to this stress is insufficient graft length, which can create undue tension at the anastomosis during body or limb movements [19]. Additionally, the use of end-to-side anastomosis increases stress concentration, particularly when a substantial size mismatch exists between the graft and the native vessel [20]. This problem is further aggravated by the use of excessively rigid prostheses, which fail to accommodate the natural pulsatile behavior of arterial walls. Each heartbeat generates a pulse wave, causing the arterial wall at the anastomotic site to expand approximately 10% more than the prosthetic segment. This discrepancy in compliance leads to a mechanical strain focused on the weakest elements, such as the suture material and the transition zone between the graft and the artery. Over time, repetitive mechanical stresses can result in damage, particularly when compliance mismatch or improper size alignment exacerbates these effects. Such damage ultimately contributes to the formation of anastomotic pseudoaneurysms [17,19,20,21]. Studies underscore the importance of surgical techniques that minimize biomechanical incompatibility, emphasizing the need for proper graft length and flexibility. An overly rigid graft not only increases strain on the anastomotic site but also fails to adapt to physiological movements, thereby amplifying the risk of mechanical failure. Thus, ensuring better material properties and anatomical fit is critical in reducing the likelihood of pseudoaneurysm formation and improving long-term outcomes for vascular procedures [17,19,20,21].

Most anastomotic pseudoaneurysms involved the common femoral artery (50 cases, 90.91%), while pseudoaneurysms of the popliteal artery were observed in 3 patients (5.45%), and pseudoaneurysms in the superficial femoral artery or deep femoral artery were found in 1 patient (1.82% each). Similarly, in the study by Marković et al., the most common location was the common femoral artery (86.2%), followed by the popliteal artery (11.5%) [17]. The frequency of aneurysms in the common femoral artery can be attributed to it being the most frequent site for arterial anastomosis. Additionally, high blood pressure can exacerbate an existing defect and cause the pseudoaneurysm to grow. Furthermore, the inguinal ligament, due to its location, can exert pressure on the blood vessels and anastomosis sites during body movements such as hip flexion. Constant tension and movement may lead to damage to the vessel wall or the anastomotic material, weakening the structure and contributing to the development of a pseudoaneurysm [17,22]. The popliteal artery, the second most frequent site for pseudoaneurysm formation, is also exposed to hemodynamic stress. This is due to its location in the popliteal fossa and the change in its tension during limb flexion and extension. Moreover, the transition of the femoral artery into the popliteal artery through the adductor hiatus, where it is subjected to traction and compression forces, promotes turbulent flow and increases blood pressure. Additionally, proteolytic degradation of the vessel wall, resulting from impaired production and regulation of matrix metalloproteinases, is another factor predisposing the popliteal artery to aneurysm development [23]. However, it should be noted that our analysis included 55 patients, of which only 11 (20.00%) had an anastomosis for the popliteal artery, resulting in a small number of pseudoaneurysms at this location in the analyzed group.

Current data suggest that the most optimal therapeutic approach for pseudoaneurysms resulting from arterial anastomoses is aneurysmectomy with graft interposition or bypass [17,23,24]. In our cohort, this was the most commonly used treatment method (46 cases, 83.64%), which is similar to the results of previous studies regarding the treatment of para-anastomotic pseudoaneurysms, where between 52.6% and 92.42% of patients underwent these procedures [17,23,24]. Pogorzelski et al. [24] reported only 3.5% of cases treated with Endovascular Aneurysm Repair (EVAR), which aligns with our results, where only three patients (5.45%) underwent treatment with this method. EVAR is not the standard method for treating pseudoaneurysms due to the risk of recurrent perfusion of the treated pseudoaneurysm, stent-graft migration, or endoleaks, which often leads to the need for additional interventions. Furthermore, long-term data on stent graft patency are unsatisfactory, ranging from 43% to 87%, which may be attributed to repeated compressive trauma to the stent graft due to its location [25,26,27,28,29,30].

Early postoperative complications were observed in 9 patients (16.36%). Acute limb ischemia occurred in three patients (5.45%). Incidences of this complication are similar to data reported in the literature, which ranges from 1.5% to 14.44% [11,12,22]. The occurrence of acute limb ischemia may be associated with the fact that in our study 47.27% of patients had generalized atherosclerosis, and 54.55% reported a history of smoking. Substances in cigarette smoke induce endothelial cell dysfunction, smooth muscle cell remodeling, and phenotypic modification of macrophages through various molecular mechanisms. These pathological changes underlie the development and progression of peripheral vascular disease [31]. This factor, combined with impaired blood flow due to generalized atherosclerosis, can contribute to the incidence of acute lower-limb ischemia episodes.

Surgical site infection was diagnosed in three patients (5.45%), which is consistent with data from the literature, where the incidence ranges from 3.63% to 12% [22,32]. Myocardial infarction occurred in two patients (3.64%), which falls within the range reported in the literature—0.9% to 8% [11,32]. Hematoma was observed in two patients (3.64%), also in line with available data, where the incidence of this complication ranges from 3.35% to 12% [4,32].

In the present study, late postoperative complications included four cases (7.27%) of acute limb ischemia, four cases (7.27%) of graft infections, four cases (7.27%) of pseudoaneurysm recurrence, two cases (3.64%) of chronic limb ischemia, and two cases (3.64%) of myocardial infarction. Similar findings have been reported in other studies: Perini et al. observed acute limb ischemia in 7.41% of cases [33], Pogorzelski et al. reported chronic limb ischemia in 3.3% of patients [34], and Garcia et al. identified myocardial infarction in 3.8% of patients [35]. These late complications may be linked to previously discussed factors, particularly continued smoking in some patients, despite surgical intervention and thorough education on its risks. Both smoking and generalized atherosclerosis contributed to the progression of peripheral vascular disease and the increased incidence of ischemic episodes.

Graft patency 12 months after treatment for femoral artery anastomotic aneurysms was 87.23%, which is consistent with the literature, where the five-year graft patency rate following open repair of femoral artery aneurysms remains at 85% [8,11,33]. According to the study by Perini et al., the presence of diabetes and ischemia are significant risk factors for late graft thrombosis [33]. Additionally, smoking is an independent risk factor for subacute stent thrombosis in patients with acute myocardial infarction [36]. The patients described in this study exhibited these risk factors for graft thrombosis. It is also important to consider the anatomical location of the femoral artery near the inguinal ligament, which, during hip flexion, causes repeated pressure on the graft during walking.

### Limitations of the Study

As with all retrospective studies, our analysis has several limitations. It was conducted on a selected, small cohort of patients from a single medical institution, which may limit the generalizability of the results. Additionally, the under-representation of women in the study cohort raises concerns about the extrapolation of findings to a broader female population. Another important factor that may have influenced the results was the limited access to healthcare during the COVID-19 pandemic, which could have delayed the diagnosis of aneurysms in the years 2020–2022 and impacted the clinical presentation of patients.

## 5. Conclusions

This study highlights the critical importance of regular follow-up in patients who have undergone surgical bypass for PAD, as para-anastomotic aneurysms can develop many years after the initial procedure. This study also emphasizes the complexity of managing lower-limb para-anastomotic pseudoaneurysms, noting a predominance of male patients and a wide range of clinical presentations, with lower-limb pain being the most common symptom. Aneurysmectomy with graft interposition was the most commonly used therapeutic approach, demonstrating its effectiveness as a primary intervention in this patient population. Most pseudoaneurysms were found in the common femoral artery, likely due to the frequency of arterial anastomosis and the mechanical stress placed on this site. Long-term outcomes showed favorable graft patency rates and low long-term mortality. Overall, our findings underscore the need for vigilant monitoring and personalized interventions in patients with para-anastomotic pseudoaneurysms to optimize clinical outcomes.

## Figures and Tables

**Table 1:** Patients’ descriptive and demographic data.

Variable	n (%), Mean/Median (Range, SD/IQR)
Age (years)	67.45 (54–83, SD: 6.89)
Gender	
Male	41 (74.55%)
Female	14 (25.45%)
Presence of comorbidities (yes)	49 (89.09%)
Arterial hypertension	38 (69.09%)
General atherosclerosis	26 (47.27%)
Coronary artery disease	19 (34.55%)
Diabetes mellitus	15 (27.27%)
History of myocardial infarction	13 (23.64%)
Dyslipidemia	11 (20.00%)
Heart failure	7 (12.73%)
COPD	1 (1.82%)
Dialysis dependence	1 (1.82%)
History of cigarette smoking (yes)	30 (54.55%)
Current cigarette smoking (yes)	21 (38.18%)
Clinical symptoms (yes)	34 (61.82%)
Lower-limb pain	19 (34.55%)
Intermittent claudication	9 (16.36%)
Ulcer	4 (7.27%)
Aneurysm rupture	4 (7.27%)
Acute limb ischemia	3 (5.45%)
Gangrene	2 (3.64%)
Limb swelling	2 (3.64%)

Abbreviation: COPD—chronic obstructive pulmonary disease, SD—standard deviation, and IQR—interquartile range.

**Table 2:** Anastomotic aneurysm characteristics.

Variable	n (%), Mean/Median (Range, SD/IQR)
Primary anastomosis	
Aortobifemoral bypass	36 (65.46%)
Femoropopliteal bypass	11 (20.00%)
Iliofemoral bypass	5 (9.09%)
Aortofemoral bypass	3 (5.45%)
Time after primary procedure to diagnosis of anastomotic aneurysm (years)	8.78 (0.21–25.24, IQR: 8.70)
Time after primary procedure to diagnosis of anastomotic pseudoaneurysm (time groups)	
Less than 1 year	2 (3.64%)
1–5 years	7 (12.73%)
6–10 years	27 (49.09%)
More than 10 years	19 (34.55%)
Anastomotic pseudoaneurysm localization	
Common femoral artery	50 (90.91%)
Popliteal artery	3 (5.45%)
Superficial femoral artery	1 (1.82%)
Deep femoral artery	1 (1.82%)
Pseudoaneurysm size (mm)	38 (18–260, IQR: 24)

Abbreviations: SD—standard deviation, IQR—interquartile range.

**Table 3:** Procedural and postoperative characteristics in patients with anastomotic aneurysms.

Variable	n (%), Mean/Median (Range, SD/IQR)
Duration of procedure (minutes)	150 (55–365, IQR: 60)
Type of procedure	
Aneurysmectomy with graft interposition	46 (83.64%)
Aneurysmectomy with extra-anatomical bypass	6 (10.91%)
EVAR	3 (5.45%)
Intraoperative blood loss	
<400 mL	52 (94.55%)
>400 mL	3 (5.45%)
Transfusion of red blood cells	2 (3.64%)
Transfusion of fresh frozen plasma	1 (1.82%)
Graft material	
Synthetic	47 (85.45%)
Patient’s vein	4 (7.27%)
Non-reversed saphenous vein	3 (5.45%)
In situ saphenous vein	1 (1.82%)
Duration of hospitalization (days)	8 (4–100, IQR: 4)
Duration of hospitalization after procedure (days)	4 (2–90, IQR: 4)
Postoperative complications (yes) *	9 (16.36%)
Acute limb ischemia	3 (5.45%)
Surgical site infection	3 (5.45%)
Myocardial infarction	2 (3.64%)
Hematoma	2 (3.64%)
Reoperations	5 (11.11%)

Footnote: * One patient had more than one complication. Abbreviations: EVAR—endovascular aneurysm repair, SD—standard deviation, and IQR—interquartile range.

**Table 4:** Long-term outcomes after the anastomotic aneurysm treatment.

Variable	n (%), Mean/Median (Range, SD/IQR)
Follow-up time (months)	16.5 (1–67, IQR: 33)
Late complications *	17 (30.91%)
Acute limb ischemia	4 (7.27%)
Graft infection	4 (7.27%)
Anastomotic aneurysm recurrence	4 (7.27%)
Chronic limb ischemia	2 (3.64%)
Myocardial infarction	2 (3.64%)
Late reoperations	10 (18.18%)
Mortality	6 (10.91%)

Footnote: * One patient had more than one complication. Abbreviations: SD—standard deviation, IQR—interquartile range.

## Data Availability

The original contributions presented in this study are included in the Supplementary Material. Further inquiries can be directed to the corresponding author.

## Supplementary Materials

The following supporting information can be downloaded at: https://www.mdpi.com/article/10.3390/biomedicines12122727/s1, S1: Raw data presented in the study.
